# Copper Nanoparticles Induced Genotoxicty, Oxidative Stress, and Changes in Superoxide Dismutase (SOD) Gene Expression in Cucumber (*Cucumis sativus*) Plants

**DOI:** 10.3389/fpls.2018.00872

**Published:** 2018-07-16

**Authors:** Kareem A. Mosa, Mohamed El-Naggar, Kalidoss Ramamoorthy, Hussain Alawadhi, Attiat Elnaggar, Sylvie Wartanian, Emy Ibrahim, Hala Hani

**Affiliations:** ^1^Department of Applied Biology, College of Sciences, University of Sharjah, Sharjah, United Arab Emirates; ^2^Department of Biotechnology, Faculty of Agriculture, Al-Azhar University, Cairo, Egypt; ^3^Department of Chemistry, College of Sciences, University of Sharjah, Sharjah, United Arab Emirates; ^4^Environmental and Chemical Biology Research Group, Research Institute of Science and Engineering, University of Sharjah, Sharjah, United Arab Emirates; ^5^Center of Advanced Materials Research, Research Institute of Sciences and Engineering, University of Sharjah, Sharjah, United Arab Emirates

**Keywords:** phytotoxicity, copper nanoparticles, hydrophonic system, XRF analysis, genotoxicity, *Cucumis sativus*

## Abstract

With the increased use of metal nanoparticles (NPs), their access to the food chain has become a main concern to scientists and holds controversial social implications. This research particularly sheds light on copper nanoparticles (CuNP), as they have been commonly used in several industries nowadays. In this study, we investigated the phytotoxicity of CuNP on cucumber (*Cucumis sativus*) plants grown hydroponically. Atomic Absorption Spectroscopy (AAS), X-Ray Fluorescence (XRF), and Scanning Electron Microscopy (SEM) analysis confirmed that *C. sativus* treated with CuNP accumulated CuNP in the plant tissues, with higher levels in roots, with amounts that were concentration dependent. Furthermore, genotoxicity was assessed using Random amplified polymorphic DNA (RAPD) technique, and our results showed that CuNP caused genomic alterations in *C. sativus*. Phenotypical, physiological, and biochemical changes were assessed by determining the CuNP treated plant’s total biomass, chlorophyll, H_2_O_2_ and MDA contents, and electrolyte leakage percentage. The results revealed notable adverse phenotypical changes along with decreased biomass and decreased levels of the photosynthetic pigments (Chlorophyll a and b) in a concentration-dependent manner. Moreover, CuNP induced damage to the root plasma membrane as determined by the increased electrolyte leakage. A significant increase in H_2_O_2_ and MDA contents were detected in *C. sativus* CuNP treated plants. Additionally, copper-zinc superoxide dismutase (Cu-Zn SOD) gene expression was induced under CuNP treatment. Overall, our results demonstrated that CuNP of 10–30 nm size were toxic to *C. sativus* plants. This finding will encourage the safe production and disposal NPs. Thus, reducing nano-metallic bioaccumulation into our food chain through crop plants; that possesses a threat to the ecological system.

## Introduction

Nanotechnology, being a revolutionary science, has been a top trend field in the scientific community because of the enormous uses and utilization of nanoparticles (NPs) in many industrial sectors and research fields. NPs have been used in various domains including medicine, electronics, and cosmetics (Roco, 2003; Biswas and Wu, 2005; Nowack and Bucheli, 2007). Despite the obvious benefits that NPs offer, there are open questions as to how the NPs used for everyday life may affect the environment. The extensive utilization of these NPs allows them to be unintentionally released into the environment either during production, use or disposal (Gottschalk and Nowack, 2011; Peralta-Videa et al., 2011; Rico et al., 2011). Increased production of NPs leads to toxic effects on living organisms including plants (Bhatt and Tripathi, 2011). Eventually, phytotoxicity in crop plants leads to threats to human health through the food chain (Servin et al., 2013). Although several reports representing phytotoxicity of NPs in plants have been demonstrated (Dreher, 2004; Yang and Watts, 2005; Wiesner et al., 2006; Handy et al., 2008; Wang et al., 2012; Yang et al., 2015), comprehensive knowledge is still lacking.

According to the United States Environmental Protection Agency (USEPA), engineered NPs are divided into four categories; carbon-based materials, metal-based materials, dendrimers, and composites (USEPA, 2007). NPs that have both positive and negative impacts on higher plants have been reported. For example, SiO_2_ and TiO_2_ enhanced the absorbance of water and fertilizers in soybean (*Glycine max*) by increasing the level of nitrate reductase enzyme to stimulate antioxidant system (Lu et al., 2002). *Pisum sativum* plants (green peas) treated with ZnO NP showed induced root growth compared to control plants (Mukherjee et al., 2014). A particular concentration of TiO_2_ NP has been shown to increase the growth of spinach by increasing photosynthesis and nitrogen metabolism (Hong et al., 2005a,b; Zheng et al., 2005; Yang et al., 2006). On the other hand, inhibition of root elongation of corn, cucumber, soybean, cabbage, and carrot was demonstrated when plants were treated with nanoscale alumina (nano-Al_2_O_3_) powders (Yang and Watts, 2005). To the best of our knowledge, this study by Yang and Watts, 2005 was the first study which highlighted the negative impact of metal NPs in plants and initiated the focus on studying the phytotoxic effect of metal NPs on plants. Later, several reports investigated the phytotoxic impact of several NPs in different plant species. For example, maize seedlings, treated with TiO_2_ NP showed reduced leaf growth and transpiration (Asli and Neumann, 2009).

Copper nanoparticles (CuNP) received great interest in the textile industry due to their immediate antimicrobial effects when introduced into synthetic and natural fibers. Also, it is now being used in the microelectronics industry for its highly conductive properties where it is being implied to conductive pastes and heaters (Ravishankar Rai and Jamuna Bai, 2011). Copper oxide nanoparticles (CuONP) have been used as catalysts for a typical C-N cross-coupling reactions (Pan et al., 2011; Poreddy et al., 2015). Copper oxide is a semiconductor with special optical, electrical, and magnetic properties; it has been used in developing super capacitors and sensors (Dagher et al., 2014; Devi et al., 2014; Zhang et al., 2014). Due to its antimicrobial and biocidal activities, CuONP attracted more researchers than other metal NP for medical applications (Perreault et al., 2012; Nations et al., 2015). In the medical field, CuONP are used to detect viruses in the human body (Ahamed et al., 2014). Sankar et al. (2014) highlighted the anticancer activity of CuONP on human lung cancer cells and showed apoptosis due to reactive oxygen species (ROS) dependent disruption of mitochondrial membranes.

Despite their many useful applications, CuNP might also have a negative impact on the environment. Generally, the toxicity of the NPs depends on size, surface charge, and pH of the environment (Chang et al., 2012). CuNP on algae showed higher toxic effect than its bulk material and the existence of the toxicity remains after 72 h of the treatment (Aruoja et al., 2009). Mussels *Mytilus galloprovincialis* treated with CuONP exhibited higher production of ROS which leads to genotoxicity and cancer (Ruiz et al., 2015). Additionally, potential toxic effects of CuONP have been reported in different tissues in rainbow trout (*Oncorhynchus mykiss*). (Baek and An, 2011; Isani et al., 2013; Ostaszewska et al., 2016).

Phytotoxicity of CuNP has been studied on wheat and mung bean plants grown on agar media and was found to reduce seedling and shoot growth (Lee et al., 2008). Additionally, Kim et al., 2012 reported the phytotoxic effect of CuONP with a size of 50 nm on cucumber plants grown hydroponically which exhibited a significant increase in ROS enzymes; catalase (CAT), peroxidase (POD), and superoxide dismutase (SOD) (Kim et al., 2012). In another report, CuONP significantly reduced the growth of cucumber plants cultivated on soil microcosm system (Kim et al., 2013). Although, the previous reports suggested the phytotoxic effects of CuNP on plants, the underlying physiological, cellular, and molecular mechanisms need further investigation.

In this study, a comprehensive analysis on cucumber (*Cucumis sativus*) plants treated with CuNP with an average size of 10–30 nm in a hydroponic system has been performed. The hydroponic system was used to maintain the uniform concentration of NPs in each different treatment compared to soil system grown plants. The phytotoxicity and genotoxicity of CuNP and its accumulation in *C. sativus* plant tissues were investigated in details.

## Materials and Methods

### Characterization of CuNP

Copper nanoparticles with an average particle size of 20 nm were purchased from Hengqiu Graphene Technology (Suzhou) Co., Ltd., Shanghai, China with a purity of 99.9%. To find out the exact size and nature of the NPs, they were analyzed using SEM. The analysis was carried out using a TESCAN VEGA4 XM SEM (SE Detector, 30 kV, high vacuum). Conductive carbon tape was used to attach the sample to the measurement stub.

### Seed Germination and Seedling Development

Soil mixture was prepared by mixing autoclaved Peat Moss with Perlite in a 3:1 ratio and was then made moist by adding distilled water. The mixture was then added to sterile plastic pots. The overnight soaked seeds of *C. sativus* were placed randomly onto the surface of the pots and a pinch of soil was added on top to fully cover the seeds. The pots were placed in the growth chamber and were watered at regular intervals for 3 weeks.

### Development of Hydroponic System

*Cucumis sativus* plants of 3 weeks old were carefully removed from the soil, washed with distilled water, transferred to glass jars containing 20% Hoagland solution prepared from Hoagland’s No. 2 Basal Salt Mixture (Sigma-Aldrich), and placed in the growth chamber maintained at 25°C (12 h with light) and 15°C (12 h dark) for 3 weeks. After 7 days of growth in the hydroponic system, well-developed lateral root system was observed. The jars were replaced every 3 days with fresh Hoagland solutions to maintain stable health.

### Nanoparticles Treatment and Plant Tissues Harvesting

Various concentrations (50, 100, and 200 mg/L) of CuNP powders were dispersed in distilled water and sonicated for 30 min, and the required Hoagland powder was mixed with it prior treatment. The 6 weeks old plants were treated by replacing the Hoagland solution with fresh Hoagland solution containing CuNP and placed back in the growth chamber for 4 days. There were 8 replicate plants per treatment. After the 4 days treatment, plants were washed to remove excess particles and dried on a tissue paper for 30 min. Fresh biomass was determined for each plant before and after treatment. Shoot and root of four replicates were separated, and kept in a 50 ml polystyrene falcon tube and then stored at -80°C until further use. The remaining four replicates were kept in a paper bags and dried in the oven (65°C) till complete dryness for metal analysis.

### Metal Analysis

Oven dried tissues of shoots and roots were weighed, crushed to powder, and digested with Nitric acid (HNO_3_) at 95°C water bath till brown fumes ceased. After cooling; distilled water and 30% H_2_O_2_ were added and samples were heated till no effervescence was observed. HCl was added to the mixture and heated. The samples were allowed to cool and filtered, and then metal accumulation was measured using Atomic Absorption Spectroscopy.

### XRF and SEM Analysis of CuNP in Plant Tissues

The XRF analysis was performed using a Horiba XGT 7200 X-ray analytical microscope. The microscope is equipped with a 50-W Rhodium X-ray source (operating at up to 50 kV) and a silicon drift detector. Two beam sizes of diameter 50 μm and 1.2 mm are available for spot and mapping analysis. XRF microscopy was used for elemental analysis of *C. sativus* roots treated with 200 mg/L CuNP. Oven dried root samples treated with CuNP were ground using mortar and pestles. The powdered samples were coated with a thin layer of gold for further analysis with SEM. The SEM analysis was carried out similar to the analysis of CuNP powders described previously.

### DNA Extraction and RAPD Analysis

Root samples stored at -80°C were powdered in liquid nitrogen, and DNA extraction was performed using NORGEN BIOTEK DNA extraction kit for plant/fungi according to the manufacturer instructions. The quality of the extracted DNA was checked by gel electrophoresis on 1% agarose gel and was quantified using NanoDrop. PCR reactions were prepared using NORGEN BIOTEK CROP master mix kit according to the manufacturer’s instruction. The PCR tubes were inserted into the PCR machine, and the random amplified polymorphic DNA (RAPD) program was set (5 min initial denaturation at 94°C, 35 cycles at 95°C for 3 min, annealing for 1 min at 40°C, extension at 72°C for 2 min, and final extension set at 72°C for 7 min). The PCR products were then run at 2% agarose gel observed under UV to check for the presence or absence of bands. RAPD primers used in this experiment were OPA-07 (GAAACGGGTG), OPA-08 (GTGACGTAGG), and OPA-09 (GGGTAACGCC).

### Chlorophyll Analysis

Shoot samples were ground in liquid nitrogen. Chlorophyll a and b contents were quantified by adding 5 ml of 80% acetone to 300 mg of powder tissue samples and placed in a shaker in the dark for 15 min, then centrifuged at 4°C for 15 min at a speed of 3,000 RPM. The supernatant was transferred to a new centrifuge tube and diluted with 80% acetone (1:5 ratio). The absorbance was measured using a spectrophotometer at two wavelengths: 663 nm (chlorophyll a) and 645 nm (chlorophyll b), and chlorophyll contents were calculated.

### Electrolyte Leakage Estimation

After CuNP treatment, 0.1 g of control and treated root tips were excised and placed in falcon tubes containing 10 ml deionized water. The initial electrical conductivity (C_I_) was measured at time zero. After 48 h, C_N_ was measured. The samples were then autoclaved at 121°C, allowed to cool and the final electrical conductivity (C_F_) was measured. Three readings of the three replicates were measured. Degree of electrical leakage was calculated using the following formula: *E*_T_ = ((*C*_N_-*C*_I_)/C_F_) × 100. The experiments were performed in triplicate.

### DAB Staining

After the CuNP treatment, the plant roots were washed well with tap and distilled water to get rid of the surface attached NPs. A small portions of the roots were excised and stained with 3, 3′-diaminobenzidine (DAB) (Daudi and O’Brien, 2012). Two ml of the DAB staining solution (1 mg/ml with 10 mM Na_2_HPO_4_) were added into the portion of the root samples and kept in desiccator for 5 min to allow the DAB into the tissues. Hence the DAB is light sensitive the samples were covered with aluminum foil. The samples were kept in a shaker for 4–5 h for incubation. After the incubation, DAB solution was removed and washing solution (ethanol:acetic acid:glycerol = 3:1:1) were added and kept for 15 min at 90°C for chlorophyll removal. Then the wash solutions were changed and observed under light microscope.

### Lipid Peroxidation and H_2_O_2_ Content

Lipid peroxidation was analyzed by measuring malondialdehyde (MDA) content according to Zhou and Leul (1998). 100 mg of control and treated plant tissues were homogenized with 1 ml of 0.1% (w/v) trichloroacetic acid (TCA) then centrifuged at 10,000 rpm for 15 min. In another 15 ml Falcon tube, 2 ml of TBA (0.5%) in TCA (20%) were added along with 800 μl of the supernatant. This mixture was incubated at 80°C water bath for 30 min and then immediately cooled into ice for 5 min. Then the mixture was centrifuged for 13,500 rpm, 5 min at 4°C. The supernatant was transferred into cuvettes and measured absorbance at 532 and 600 nm after blanking. The level of MDA was represented as nmol g^-1^ FW using extinction coefficient of 155 mM^-1^cm^-1^.

For determination of H_2_O_2_ content, 0.1 g of ground tissues were mixed with 2 ml of 0.1% TCA (w/v) and then the homogenate were centrifuged at 12,000 rpm for 15 min (Velikova et al., 2000). 0.5 ml of the supernatant was mixed with 0.5 ml of potassium phosphate buffer (10 mM, pH 7) and 1 ml of 1 M potassium iodide (KI) was added and checked their absorbance at 390 nm. The H_2_O_2_ content was determined using extinction coefficient of 0.28 μm cm^-1^ and it was measured as nmol g^-1^ FW.

### RNA Extraction and Quantitative Real-Time PCR (qRT-PCR)

0.1 g of the -80°C stored control and CuNP treated plant tissues were ground in liquid nitrogen and were used to extract total RNA using Qiagen RNeasy^®^ Plant Mini kit protocol. RNA concentrations were checked using NanoDrop, and the final concentrations of 1 μg RNA were used for cDNA synthesis using Invitrogen^TM^ SuperScript^TM^ II Reverse Transcriptase according to manufacturer’s protocol. Real-time PCR was performed with the following primer pairs; copper-zinc SOD (Cu-Zn SOD)_F (GGTTGCTGGTGATGATGGTACTG), Cu-Zn SOD_R (TGCATGGACAACAATAGACCTTCC), Actin_F (GGAAATACAGTGTCTGGATTGGAG), Actin_R (TGAACTTAGAAGCACTTCCTGTG) with Power SYBR^TM^ Green PCR Master mix (Applied Biosystems^TM^) using BioRad CFX96 Touch^TM^ Real-Time PCR detection system. The real time data were analyzed using 2^-ΔΔ^*^C^*_T_ method (Livak and Schmittgen, 2001).

## Results

### Characterization of CuNP

**FIGURE 1:**
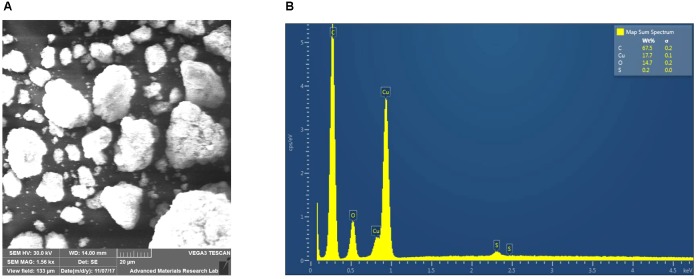
Characterization of the copper nanoparticles (CuNP). **(A)** Scanning Electron Microscope-Electron Dispersive X-ray Spectroscopy (SEM-EDS) analysis of copper (Cu) nanoparticles. **(B)** EDX characteristic spectrum shows the presence of copper, and oxygen in the sample confirms the oxidation of the CuNP by air.

SEM analysis confirmed that the purchased CuNP sizes were in the 10–30 nm range (**Figure 1A**). Scanning Electron Microscope-Electron Dispersive X-ray Spectroscopy (SEM-EDS) analysis also showed the presence of oxygen along with copper, indicating that at least part of the CuNP was oxidized by air (**Figure 1B**).

### Biomass Analysis of CuNP Treated *C. sativus* Plants

**FIGURE 2:**
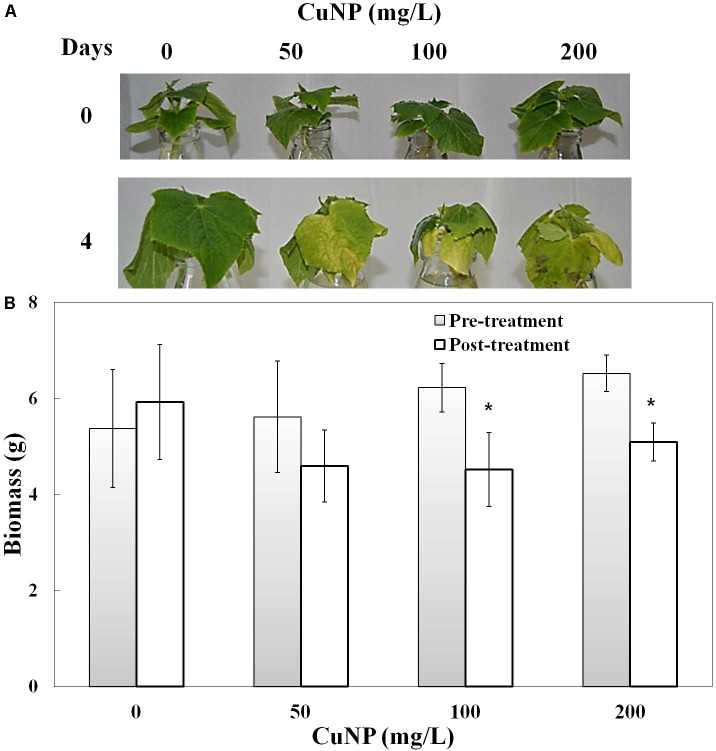
Phenotype **(A)** and biomass measurement **(B)** of *Cucumis sativus* treated with various concentrations of CuNP (50, 100, and 200 mg/L) in pre- and post-treatment. Error bars represent standard errors of mean values of eight replicates. Statistically significant difference was calculated at ^∗^*P* ≤ 0.05, ^∗∗^*P* ≤ 0.01.

Plants treated with various concentrations of CuNP showed a clear senescence effect (leaf yellowing) at the end of the treatment period (4 days), with brown spots observed compared to control plants (**Figure 2A**). The total biomass of the *C. sativus* plants was measured before and after treatment (**Figure 2B**). Biomass analysis showed significant biomass reduction of *C. sativus* plants treated with 100, and 200 mg/ L CuNP for 4 days compared with the biomass of the same plants before treatment. Although, the biomass of the plants treated with 50 mg/L CuNP showed decreased biomass as well, but it was not significant. Furthermore, as expected, the non-treated control plants (0 mg/ L CuNP) showed an enhanced but not significant increase in the biomass after the 4 days.

### Copper Detection and Accumulation

**FIGURE 3:**
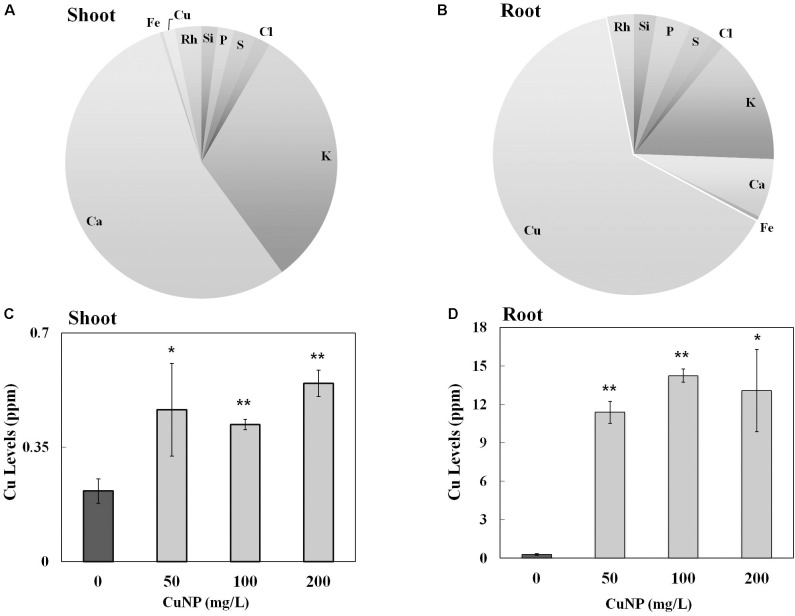
Elemental and metal analysis by XRF and AAS. Relative element concentrations in CuNP treated *C. sativus* plants represented as (mass%) in **(A)** shoot and **(B)** root tissues analyzed by XRF. Metal analysis of control and CuNP treated *C. sativus* plant shoots **(C)** and roots **(D)** analyzed by AAS. Error bars represent standard errors of mean values of three replicates. Statistically significant difference was calculated at ^∗^*P* ≤ 0.05, ^∗∗^*P* ≤ 0.01.

**FIGURE 4:**
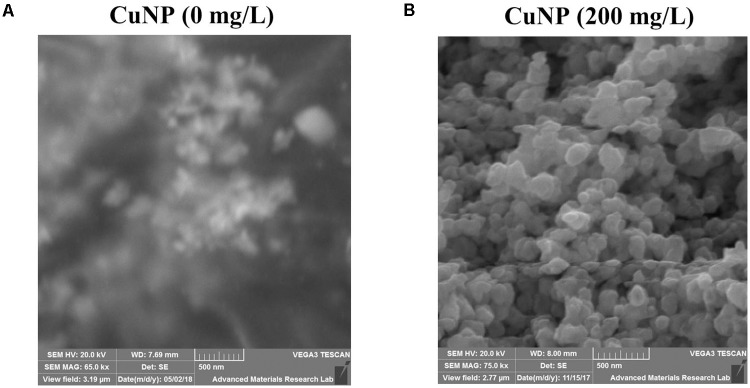
Scanning Electron Microscope (SEM) image showing **(A)** no CuNP were observed in the control roots, and **(B)** CuNP were observed in aggregates in 200 mg/L CuNP treated root samples of *C. sativus*.

To investigate whether *C. sativus* plants treated with CuNP mediate CuNP accumulation, XRF microscopy was utilized to measure the relative elemental composition represented as mass% of *C. sativus* shoot and root tissues treated with CuNP (**Figures 3A,B**). Besides copper, other elements of the Hoagland solution, such as Ca, K, Si, P, S, and Cl were also detected. Interestingly, the XRF analysis of shoots and roots exhibited more copper in roots compared to shoots (**Figures 3A,B**). To confirm and to quantify the Cu levels, the concentrations of Cu in shoots and roots of *C. sativus* plants were measured after 4 days of CuNP treatment using AAS (**Figures 3C,D**). The results showed increased Cu accumulation in these plants in proportion to the CuNP concentrations (100 and 200 mg/L). Again, it was observed that the accumulation of CuNP was more in the roots compared to the shoots. SEM analysis of *C. sativus* root samples confirmed the presence of CuNP in aggregated form and the sizes ranged between 80–140 nm in the treated plant tissues (**Figure 4**). However, no CuNP were observed in the control roots.

### Genotoxicity Analysis by RAPD

**FIGURE 5:**
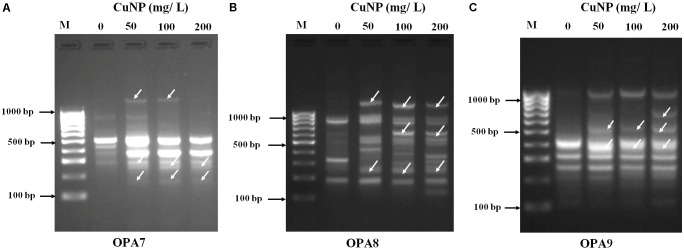
Random amplified polymorphic DNA (RAPD) analysis. Banding patterns generated with different random oligonucleotide primers **(A)** OPA7, **(B)** OPA8, and **(C)** OPA9 to evaluate the genotoxicity effects of CuNP in *C. sativus.* [Lanes M- Molecular marker (100 bp), 1- control 0, 2- 50 mg/L, 3- 100 mg/L, 4- 200 mg/L of CuNP].

Random amplified polymorphic DNA was employed to assess the CuNP toxicity effect on the genomic level of *C. sativus*. Genomic DNA was extracted from the root of both control, and CuNP treated samples. Various set of primers were used to amplify all DNA samples. Control sample amplified OPA- 07 primer showed five bands ranging between 300 to 800 bps. For 50 mg/L treatment of CuNP sample DNA amplified same bands as the control DNA sample as well as two additional bands at 200 and much above 1000 bp marker band. Similar band patterns were observed for DNA samples treated with 100 mg/L CuNP. In the case of the 200 mg/L CuNP treated DNA sample amplified same bands of control DNA with the exception of 800 bp and the additional 200 bp band (**Figure 5A**). Control sample DNA for OPA- 08 primer amplified three high intensity bands between 200 to 800 bps. For 50 mg/L treatment of CuNP sample DNA amplified several bands ranging from 300 to above 1000 bps marker band. In case of 100 and 200 mg/L treated with CuNP sample amplified almost similar band patterns of same intensity (**Figure 5B**). Control sample DNA amplified with OPA- 09 primer exhibited six bands ranging from 100 to 1000 bps. Samples treated with 50 and 100 mg/L CuNP showed two additional bands at 400 and 500 bps. However, 200 mg/L treated sample DNA amplified same band pattern as 50 and 100 mg/L treated sample DNA along with an additional band at 600 bps (**Figure 5C**).

### Effect of CuNP on Chlorophyll Content

**FIGURE 6:**
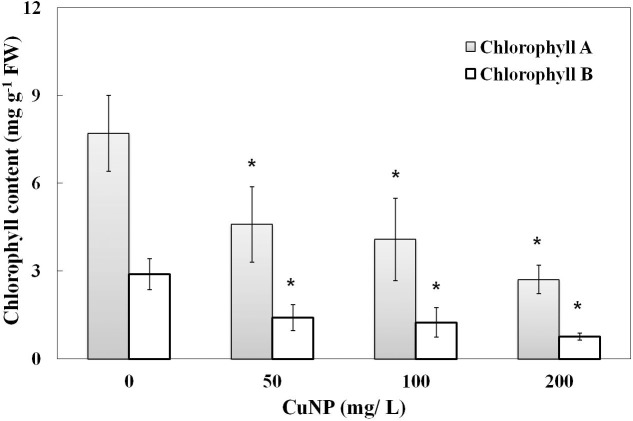
Chlorophyll content of control and CuNP treated *C. sativus* plants. Control plants have higher chlorophyll content, and when the concentration of CuNP increases the chlorophyll content decreases. Error bars represent standard errors of mean values of four replicates. Statistically significant difference was calculated at ^∗^*P* ≤ 0.05, ^∗∗^*P* ≤ 0.01.

When the plants were treated with CuNP for 4 days, induced senescence between control and treated plants was observed. To quantify the levels of senescence, the contents of chlorophyll a and b were measured. Compared to the untreated control plants, shoots of CuNP treated plants showed a significant decrease in chlorophyll a and b contents as the concentration of CuNP increased (**Figure 6**).

### Electrolyte Leakage Analysis

**FIGURE 7:**
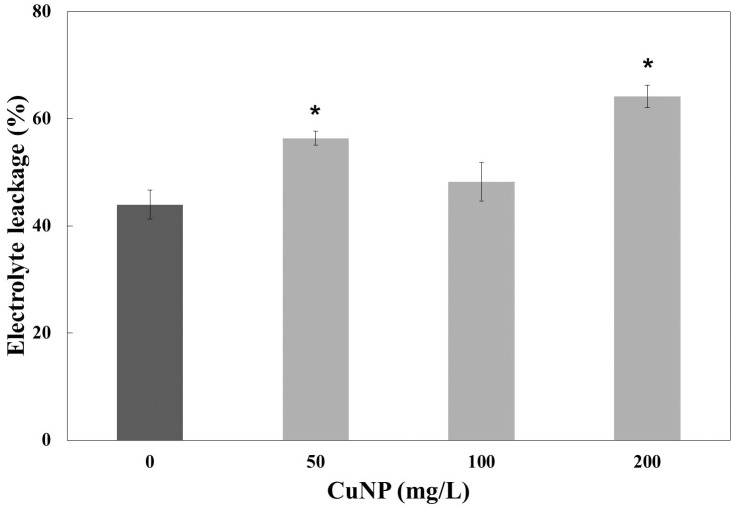
Electrolyte leakage analysis of CuNP on *C. sativus* plants. Error bars represent standard errors of mean values of three replicates. Statistically significant difference was calculated at ^∗^*P* ≤ 0.05, ^∗∗^*P* ≤ 0.01.

To measure the plasma membrane integrity of the *C. sativus* plants treated with CuNP, electrolyte leakage analysis was performed. The results showed increased electrolyte leakage in CuNP treated plants compared to control plants (**Figure 7**). Hence, CuNP induced damage to root plasma membrane of *C. sativus* as indicated by the significant increase in electrolyte leakage in 50 and 200 mg/L CuNP treatment.

### Effect of CuNP on H_2_O_2_ and MDA Contents

**FIGURE 8:**
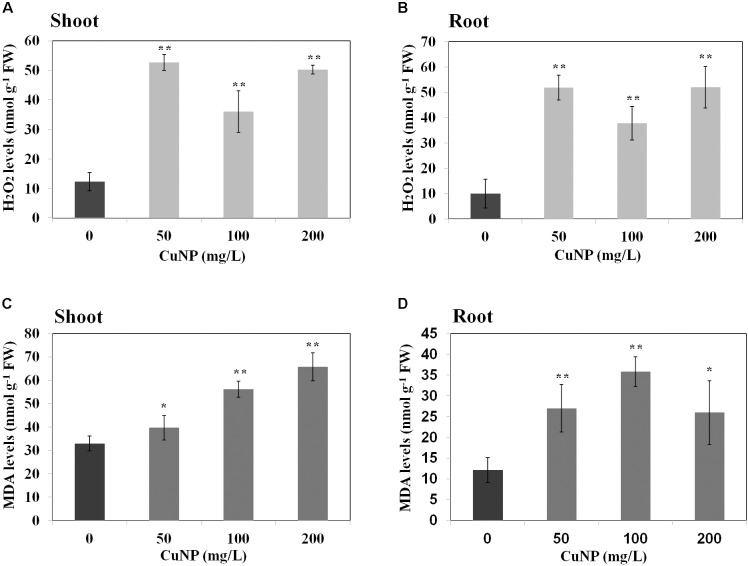
Effect of CuNP on H_2_O_2_ **(A,B)** and MDA **(C,D)** contents in *C. sativus* shoots and roots, respectively. Error bars represent standard errors of mean values of three replicates. Statistically significant difference was calculated at ^∗^*P* ≤ 0.05, ^∗∗^*P* ≤ 0.01.

To examine whether CuNP induced oxidative stress on *C. sativus* plants, H_2_O_2_ and lipid peroxidation contents were measured (**Figure 8**). H_2_O_2_ levels in both shoots and roots of all the concentrations we checked were higher compared to control plants. The levels were almost four times higher than control plants of shoots and roots (**Figures 8A,B**).

In terms of lipid peroxidation, we measured MDA levels in both control and CuNP treated *C. sativus* plants. The root and shoot samples of *C. sativus* treated plants showed significant increase in all the concentrations of CuNP as compared to the control plants (**Figures 8C,D**).

### H_2_O_2_ Detection by DAB Staining

**FIGURE 9:**
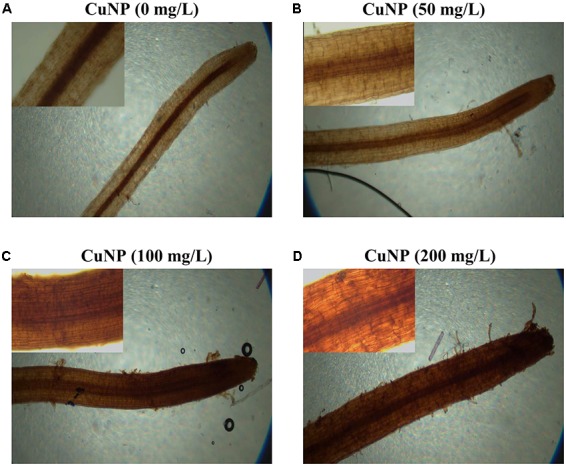
H_2_O_2_ detection in *C. sativus* root tip by DAB stain. **(A)** control, **(B)** treatment with 50 mg/L, **(C)** treatment with 100 mg/L, and **(D)** treatment with 200 mg/L. For each group of treatment the magnified image (20×) of the root tip were shown inside each panel.

To visualize the H_2_O_2_ accumulation in root tips due to CuNP treatment, we performed the histochemical staining with DAB reagent. The roots observed under the light microscope showed a clear difference between control and treated plants. CuNP treated *C. sativus* roots stained more than control plants in a dose-dependent manner as the CuNP concentration increases it induces higher H_2_O_2_ accumulation (**Figure 9**).

### Impact of CuNP on Superoxide Dismutase (SOD) Gene Expression

**FIGURE 10:**
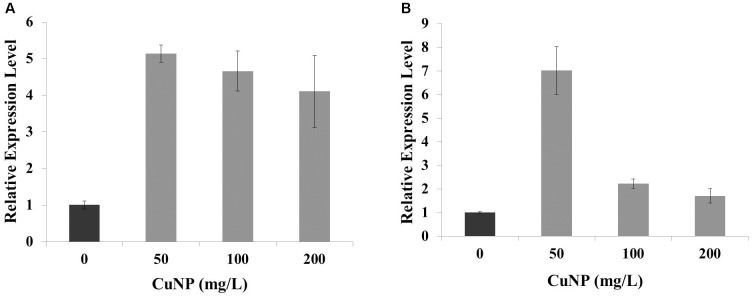
Relative expression of copper-zinc SOD (Cu-Zn SOD) gene under CuNP (50, 100, and 200 mg/L) treatment in **(A)** shoots and **(B)** roots of *C. sativus*.

Superoxide dismutase is one of the major enzymatic antioxidants. In order to evaluate the regulation of *C. sativus* Cu-Zn SOD gene in response to CuNP treatment, quantitative RT-PCR (qRT-PCR) was performed to analyze the changes in transcripts of Cu-Zn SOD in response to 50, 100, and 200 mg/L of CuNP in *C. sativus* shoots and roots (**Figure 10**). Expression levels of Cu-Zn SOD were induced in shoots under 50, 100, and 200 mg/L of CuNP compared to control untreated plants (0 mg/L of CuNP) (**Figure 10A**). Similarly, in 50 mg/L CuNP treated roots, the expression level of Cu-Zn SOD was increased by six-fold compared to control untreated roots. However, a clear decrease but still significantly higher than control untreated roots was detected in Cu-Zn SOD expression level in response to 100, and 200 mg/L of CuNP (**Figure 10B**).

## Discussion

Generally, metal NPs have really strong bonds that allow them to reside within the environment for a long time as they are hardly degraded or destroyed (Svintradze and Pidaparti, 2010). This puts the ecological system in great danger as metal NPs possess highly reactive or catalytic properties compared to their bulk material, and thus can be potentially toxic. The current research highlighted the phytotoxicity effect of CuNP on cucumber (*C. sativus*) plants under hydroponic growth conditions, due to the limited studies available concerning the implications of CuNP and its phytotoxic effect on *C. sativus* plants. We have chosen the hydroponic system for this study to obtain a uniform concentration of NPs compared to soil systems. If we perform such experiments in soil grown plants, it’s difficult to attain the uniformity in the concentration of NPs as well as the clear impact of toxicity on plants. Furthermore, for maintaining the uniformity of the NPs by growing plants on agar media, though its well suited for seedlings stage (Taylor and Foy, 1985; Munzuroglu and Geckil, 2002; Lee et al., 2008) rather than mature plants (6–7 week old in our study). *C. sativus* was selected for our study as one of the plant species recommended by McLean and Bledsoe (1996) for phytotoxicity studies. There are high chances of Cu oxidation by air to become copper oxide (Miyagawa et al., 2016). The purchased CuNP got oxidized to form copper oxide by air exposure as indicated by SEM-EDS analysis.

In the present study, we observed that plants exposed to CuNP exhibited higher toxicity based on the concentration of the CuNP with regard to its biomass reduction, which was similar to the reported earlier in hydroponically and soil grown cucumber plants treated with CuONP of 50 nm size (Kim et al., 2013). X-Ray Fluorescence (XRF) is one of the advanced techniques to estimate the relative quantity of elements present in the sample as semi-quantitatively (mass%). Earlier, Attaelmanan and Kawam, 2012 evaluated the elemental composition of *Calotropis procera* using such XRF microscope (Attaelmanan and Kawam, 2012). We used the same technique and the same instrument to show the elemental composition of *C. sativus* shoot and root samples treated with CuNP. There were several reports regarding micro XRF analysis of NPs treated plant tissues. micro XRF analysis of TiO_2_ of hydroponically grown cucumber plants showed titanium accumulation in their leaf trichomes (Servin et al., 2012). The translocation of TiO_2_ NP and multi-walled carbon nanotubes in red clover and wheat plant tissues were studied using XRF techniques (Gogos et al., 2016). Wang et al. (2017) demonstrated that phytotoxicity of silver sulfide NP in dicot and monocot plants and used XRF analysis to show the NP accumulation. Our elemental analysis of CuNP treated samples revealed 64 and 1.38% of Cu in root and shoot tissues, respectively.

To characterize the CuNP in cucumber treated plant tissues we performed Scanning Electron Microscopy (SEM) analysis. SEM analysis of CuNP treated root samples showed the aggregates of CuNP. Several reports already demonstrated the detection of NPs in aggregated form within the plant tissues regardless the small size of the NPs used for the treatment. González-Melendi et al. (2007) reported that charged NPs can be targeted into specific locations of plant tissues for delivery purposes and found that they tend to form aggregates in their targeted positions. TEM analysis of carbon-coated iron NP into *Cucurbita pepo* L plants showed the aggregates of NPs in various parts of the plants (Corredor et al., 2009). Another study revealed that green synthesized gold NP were found to increase rice germination, where the TEM analysis revealed the aggregated form of gold NP in root tissues of rice (Nji Tsi et al., 2017). Similarly, in our study SEM-EDS analysis showed the aggregation of CuNP in treated *C. sativus.*

Random amplified polymorphic DNA technique was employed in this study to check for any genetic alteration caused by CuNP. Unlike traditional PCR, RAPD technology is rapid, reproducible and does not require specific knowledge of the DNA sequence, which makes it ideal for examining and estimating the genomic variation in genotoxic studies (Atienzar et al., 1999). RAPD has been used to confirm the genotoxic effect of ZnO and CuO NP on buckwheat (*Fagopyrum esculentum*) seedlings (Comrey and Lee, 2013). Additionally, RAPD analysis of zucchini (*C. pepo*) plants treated with TiO_2_ NP exhibited DNA changes compared to the control plants (Moreno-Olivas et al., 2014). Genotoxicity of CeO_2_ and ZnO NP on soybean seedlings was demonstrated by the appearance of new DNA bands through RAPD analysis by López-Moreno et al. (2010). Furthermore, the genotoxicity of nCeO_2_ and nTiO_2_ NP were reported in seedlings of *Hordeum vulgare* L. using RAPD approach (Marchiol et al., 2016). In our investigation, three standard primers (OPA7, OPA8, and OPA9) were employed, and different bands appeared/disappeared when comparing the treated plants to the untreated control plants, rendering CuNP toxic to *C. sativus* at high exposure concentrations. The presence of these new bands may exhibit alterations in the priming sites leading to new annealing conditions in addition to homologous recombination which lead to the appearance of new bands (Atienzar and Jha, 2006). Our plants were more sensitive to the effects of CuNP as more band alterations were observed when compared to the control. The only difference between the treated and control plants was the presence or absence of CuNP which support that the changes in the DNA were caused by the effect of the CuNP.

The assessments of chlorophyll content of plants exposed to NPs were included as new parameters for phytotoxicity analysis (Ruttkay-Nedecky et al., 2017). When NPs are adsorbed to the root surface, it interrupts the absorption of macro and micronutrients required for plant’s development reducing the nutrient uptake and thus resulting in lower chlorophyll content. Martinez-Fernandez et al. (2016) investigated the uptake of water contaminated with iron oxide NP by the roots of *Helianthus annuus*. They found that macronutrients such as Ca, K, Mg, and S reduced subsequently in the plant’s shoot, and accordingly the chlorophyll pigmentation contents were decreased as well (Martinez-Fernandez et al., 2016). Our study has also demonstrated the negative effect of CuNP on chlorophyll a and chlorophyll b contents of *C. sativus*. The decrease in chlorophyll contents was obvious to the eyes after assessing treated and untreated plants.

The plasma membranes permeability was analyzed by testing the electrolyte leakage of the control and CuNP treated root tissues. It has been reported that 500 mg/L cerium oxide (nCeO_2_) NP increased electrolyte leakage in the roots of rice seedlings (Hernandez-Viezcas et al., 2013). Additionally, a significant increase in electrolyte leakage was reported in asparagus lettuce root cultured in an agar medium treated with cerium oxide NP (Cui et al., 2014). In our study, *C. sativus* was sensitive to CuNP and significantly increased electrolyte leakage which caused the high reactivity of CuNP. CuNP changed the permeability of the plant membrane and as a result, damaged the plant’s cell membranes which increased the probability of NPs entering into the cells. *C. sativus* showed increased permeability when plants were treated with CuNP which could be then accumulated into the roots and could also be translocated to the shoots, thus affecting the cell metabolic pathway. It has been already reported that CuONP is able to be translocated from roots to shoots (Shi et al., 2014). Our results demonstrated that CuNP were highly accumulated in roots with slight accumulations in the shoot system, indicating that only a small quantity of CuNP was translocated to the shoot of *C. sativus* during the treatment time period of our experiment (4 days). Kim et al. (2012) reported the increased accumulation of Cu and Zn ions in roots of *C. sativus* when treated with CuO and ZnO NP. It’s already reported that plant systems are tend to absorb CuONP according to their concentration in the nutrient solution (Shi et al., 2014). Plants with more thin roots accumulate more metals than those with less thick roots as the surface area is decreased. It was apparent that our *C. sativus* plants had numerous thin and long root systems resulting in higher root accumulation of Cu.

It has been already highlighted earlier that metal toxicity in plants lead to enhanced production of ROS which is causing oxidative stress by increased electrolyte leakage, protein oxidation, lipid peroxidation, DNA damage and finally cell death (Meriga et al., 2004; Sharma et al., 2012; Wani et al., 2018). Cu is catalyzing the overproduction of ROS, such as H_2_O_2_ by Haber-Weiss and Fenton reactions leading to lipid peroxidation, damaging nucleic acids, and oxidizing proteins (Gill and Tuteja, 2010; Fidalgo et al., 2013). Hence we measured the major molecules associated with oxidative stress such as MDA and H_2_O_2_ in *C. sativus* plants upon CuNP treatment. The highest levels of MDA were observed in *C. sativus* shoots and roots treated with 100 and 200, and 50 and 100 mg/L CuNP, respectively. The increase in MDA levels is directly proportional to the concentration of the CuNP used for the treatment. Similarly, such effect has been already shown in rice seedlings treated with CuONP which induced MDA levels compared to control plants (Wang et al., 2014). Likewise, the major reactive oxygen molecule, H_2_O_2_, levels were measured in control and CuNP treated plants. Our results demonstrated that all the treatment conditions showed increased H_2_O_2_ levels in both roots and shoots samples. Such induction of oxidative stress mediated H_2_O_2_ levels were also confirmed by DAB staining of root tips where dark brown stains were observed in all the treated root tip samples. Similarly, TiO_2_NP treated *Hydrilla verticillata* showed increased H_2_O_2_ which was due to oxidative stress caused by the NPs (Spengler et al., 2017).

In order to reduce the oxidative stress, plants develop a defense mechanism involving the antioxidant enzymes such as SOD to scavenge excessive ROS. SODs are classified into three isoforms; Cu-Zn SOD, manganese SOD (Mn SOD), and iron SOD (Fe SOD) based on their metal cofactors (Alscher et al., 2002). In general, SOD is catalyzing the disproportionating superoxide anion free radicals into hydrogen peroxide and molecular oxygen (Gill and Tuteja, 2010). Our results showed a significant upregulation of Cu-Zn SOD gene expression in roots and shoots of CuNP treated *C. sativus* plants, demonstrating the increased ROS production and the conversion of superoxide anion free radicals into hydrogen peroxide which is displayed in our study by the increased H_2_O_2_ levels in both root and shoot samples of CuNP treated *C. sativus*. Similarly, a significant increase in SOD gene expression was reported in *P. sativum* L roots treated with 100, 200, 400, and 500 mg dm^-3^ CuONP (Nair and Chung, 2015).

## Conclusion

This study was performed to assess the phytotoxicity of CuNP on *C. sativus*. We investigated the toxicity on the physiological, phenotypical, biochemical, and genomic levels. It was found that tested CuNP of the size 10–30 nm were toxic to *C. sativus*. CuNP showed a decrease in the total biomass of the *C. sativus* treated plants. XRF, Scanning Electron Microscope (SEM), and Atomic Absorption Spectroscopy (AAS) analysis demonstrated that CuNP were accumulated in the *C. sativus* plant tissues, with higher accumulation level in root tissues. RAPD PCR analysis confirmed the genotoxic effect of CuNP which induced genomic DNA modifications in *C. sativus*. Additionally, CuNP showed a significant decrease in chlorophyll a and b contents, increase in H_2_O_2_ and MDA contents, as well as an increase in electrolyte leakage which induced damage to cucumber root plasma membrane. Collectively, this demonstrated that CuNP induced oxidative stress in *C. sativus*. Finally, Cu-Zn SOD gene expression was induced under CuNP treatment. The availability of *C. sativus* genome information could enhance our understanding of CuNP phytotoxicity through deep molecular and gene expression analysis studies.

## Author Contributions

KM conceived and designed the experiments. KM, KR, AE, SW, EI, and HH performed the experiments. KM, ME-N, and KR analyzed the data. ME-N performed the AAS metal analysis. HA performed the XRF and SEM analysis. KM wrote the manuscript. KM, ME-N, KR, and HA edited the manuscript.

## Conflict of Interest Statement

The authors declare that the research was conducted in the absence of any commercial or financial relationships that could be construed as a potential conflict of interest.
